# Municipal waste landfill as a source of polychlorinated biphenyls releases to the environment

**DOI:** 10.7717/peerj.10546

**Published:** 2021-01-15

**Authors:** Marta Gabryszewska, Barbara Gworek

**Affiliations:** Institute of Environmental Protection - National Research Institute, Warsaw, Poland

**Keywords:** Polychlorinated biphenyls, Municipal waste landfill, Sludge, Groundwater

## Abstract

This study aimed to investigate the impact of municipal waste landfill on polychlorinated biphenyls (PCBs) release to the environment concerning groundwater flow directions. The contents of polychlorinated biphenyls in soils, plants and water were analysed at various distances from the landfill. Thanks to low solubility PCBs in water groundwater flow direction, under the landfill, have an influence on PCBs concentration in groundwater. Strong PCBs’ sorption to organic matter caused that no affect groundwater flow directions on PCB content in soils and plants’ tissues was observed. The largest PCBs deposition zone was located 50 m from the contamination source (landfill). Tri-CB and tetra-CB homologues were capable of migration deep into the soil profile, which could be related to the geological material from which the soils under study were developed, as well as to the properties of the PCB homologues.

## Introduction

Polychlorinated biphenyls (PCBs) are contaminants, which are persistent, lipophilic and hydrophobic, with a high potential for bioaccumulation in living organisms (Kaya et al., 2018). 48% of PCBs’ production, were used in transformer cooling oils and 21% in dielectric fluids of capacitors (Whylie & Warmuth, 2010). Also, PCBs were used in hydraulic and heat transfer fluids, pesticides and as lubricants in oils or greases, preservatives and impregnating agents (Kodavanti, 2017). PCBs may also be produced accidentally like during waste incineration, ferrous and non-ferrous metal production. High levels of PCBs were found as impurities in chlorinated products or as by-products in solvent production (Liu et al., 2018). It was found that PCB 11 may be formed during pigment production and can be released into the atmosphere during waste water treatment (Vorkamp, 2016). PCBs are also unintentional by-products from manufacturing of polymer resins which may have implications for relevant waste streams (Herkert, Jahnke & Hornbuckle, 2018).

Accumulation of PCBs in the soil may cause long-term contamination. Li et al. (2018) carried out experiments that consisted of examining the evaporation of PCBs from soil and water surface into the air, PCBs retention by plants, and then PCBs deposition. It was shown that the tri-CB, tetra-CB and penta-CB homologues were the dominating compounds of volatilisation and deposition (Li et al., 2018). PCBs released into the environment as a part of transformer oil spill was concentrated mainly in two compartments: soil (92.7%) and groundwater (Asif & Chen, 2016).

PCBs may be degraded in the biological way—the formation of OH-PCB (major metabolite) and MeO-PCB (minor metabolite) was found in *Bacillus subtilis* after exposure to PCBs (Sun, Pan & Zhu, 2018). In the research on polychlorinated biphenyl dechlorination by electrical stimulation in sediments, it was found that bacteria, including *Dehalogenimonas, Dehalobacter, Sulfuricurvum, Dechloromonas,* and *Geobacter*, were responsible for PCBs dechlorination (Yu et al., 2017). The fate and behaviour of PCBs in soils and their bioaccessibility can be influenced by the soil physicochemical characteristics, the concentration and properties of PCBs and their residence time in soils (Bielská, Šmidová & Hofman, 2013; Stella et al., 2015). Low solubilities of PCBs in water (ranged from 9.3 to 7.6*10^−4^ g m^−3^) allow stating that small amounts of PCBs can be expected in ground and surface water (Erickson, 2001). High octanol-water partition coefficients of PCBs (log K_ow_ ranged from 4.3 to 8.3) indicate the affinity of PCBs for the organic matter which can affect the high PCBs contents in soil (Erickson, 2001). The PCBs diffusion into soil pores and partitioning with soil organic matter (Yu et al., 2018) with prolonged time is dominant and leads to the decrease of bioaccessibility of PCBs (Ti et al., 2018).

It is well known that water has a significant impact on the transfer of pollutants. It must be pointed out that near to the landfill, wells are used as drinking water supply. Therefore, research was carried out on the release of PCBs from the 40-year old landfill to demonstrate whether there is a risk of contamination of drinking water by PCBs. At a municipal landfill, anaerobic decomposition of organic waste with the production of CO_2_ and CH_4_ occurs continuously (Murphy et al., 1985). These gases can be a carrier of pollutants such as PCBs,causing soil and water contamination as a result of deposition in the surroundings of landfills. In Illinois and Wisconsin, air samples were taken from six municipal landfills. Average PCB concentration per m^3^ methane was 285 ng PCB/m^3^ CH_4_(Murphy et al., 1985). In Norwegian waste handling facilities waste were tested for PCBs content. Sums of seven PCBs (PCB-28, -52, -101, -118, -138, -153 and -180) in plastic waste was 3700 ± 1800 µg/kg and in electrical waste and electronic equipment 1300 ± 400 µg/kg (Arp et al., 2020) It was also noted that PCBs in the air was mostly carried by the gas phase than by particles (Arp et al., 2020).

The purpose of the work was to examine the impact of the municipal waste landfill, as a source of PCBs released into the environment, on the PCBs contamination of soils, plants and water, considering the distance from the landfill. The assessment was based on spatial and profile distribution of PCBs in soils and waters from the first aquifer level in the landfill vicinity as well as on the PCBs contents in mono- and dicotyledonous plants from the area where the soil samples were taken. The impact of landfill on PCBs contents in the environment was assessed by calculating biological indices: Bioaccumulation Coefficient (BAC), Mobility Ratio (MR). Biological indices can be helpful in the assessment of pollution by PCBs, accumulation and interaction of PCBs in the environment, as well as their mobility and translocation. The BAC indices represent the substance ability to accumulate in plant. If values of calculated indices are high then this substance can accumulate in a plants. The MR indices show how much PCBs migrate from soil to groundwater. The high values of coefficients point out an insignificant impact of the PCBs contents in soils on water pollution.

The work brings new knowledge, hitherto not recognized in the literature, regarding the comprehensive environmental impact of PCBs released from the municipal waste landfills.

## Materials & Methods

### Features of the Łubna landfill

The municipal waste landfill at Łubna is located approximately 35 km south from Warsaw, Poland (52°1′49″N, 21°8′56″E). The total landfill area takes 39.63 ha, while approximately 22 ha have been used for the landfill and technical facilities. The municipal waste disposal at the landfill started in 1978 without any prior preparation of the site. Currently, the landfill stores approximately 5.5 million m^3^ of waste. They contain up to 50% an organic matter of biological origin (including more than 30% of the total mass taken by plant-based food waste), while the remaining 50% are non-biologic. Sewage sludge and hazardous waste from industrial and service facilities were stored in the landfill. A vertical anti-filtration shutter was installed around the landfill in 1998 to prevent the transfer of leachate from the landfill to the first aquifer and into drainage ditches.

**Figure 1:**
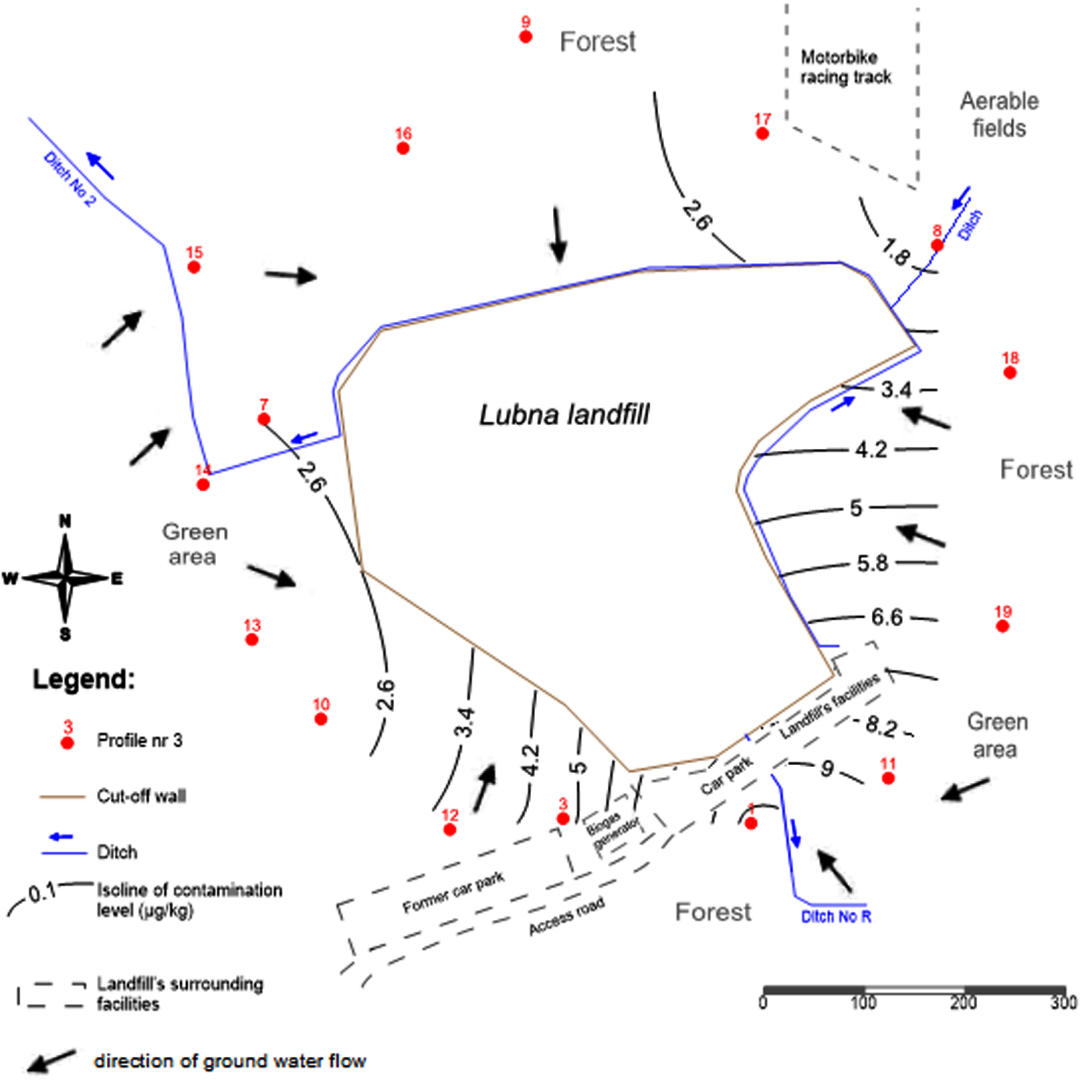
Spatial distribution of PCBs contents in the 0–20 cm soil layer around the landfill.

The landfill is an above ground efacility with a storage height of around 60 m above ground level, situated at the local drainage basin. The water outflow takes place following the area slope falling to the south, east and west, where there are local seasonal wetlands. Local drainage ditches make up the hydrographic network in the area. The main collecting ditch is ditch No. 2 (Fig. 1).

The analysis of the geological structure of the Łubna municipal waste landfill shows that its subgrade is stratigraphically and geologicaly diverse. The Łubna waste landfill is located within the Warsaw Plain, in the edge zone of the upland with terraces overlooking the Vistula. There are two main geomorphological units in the area of the landfill and its immediate vicinity: post-glacial upland, denuded and undenuded; and river valleys (Gworek et al., 2016; Koda, Pachuta & Osinski, 2013). Top layers of soils to a depth of about 2 m were built predominantly from loose sands and loamy sand.

Institution: “Municipal waste landfill Łubna”, Eugeniusz Koda, has given oral consent for sampling. Nowadays this landfill is closed and after remediation.

### Sampling

The samples were taken in the autumn after growing season. 10 study plots of an area of 10 m^2^ were designated around the landfill site. The study plots were selected according to the water flow, presence of piezometers and plants. Soil samples (mostly sandy and sandy-loam) were taken from three layers: 0–0.2, 0.2–0.5 and 0.5–0.8 m, with each sample being an average of mixed 12 subsamples for the top layer (sampled diagonally) and of 4 subsamples for the deeper layers.

The following plants were sampled for analyses from the designated study plots: *Solidago canadensis* (leaves, stem) - in the objects: 3, 7, 8,10; *Dryopteris* sp. (leaves) - in the objects:1, 8, 9; *Poaceae* - in the objects: 1, 3, 7, 8, 9, 10. Leaves covered the cuticle layer containing lipid substances which may increase the uptake of PCBs from the air. Plants were not washed. Soil and plant samples were transported to the laboratory in paper bags at room temperature on the same day.

Groundwater was taken from the first aquifer levels from a depth of 0.25–0.75 m (objects: 1, 3, 7, 8, 9, 10), and surface water was sampled from the ditch surrounding the landfill (object 2 in Fig. 1). Water samples were transported to the laboratory in glass bottles about the volume of 1 L at room temperature on the same day.

### General soil analysis

The soils for analyses were air-dried at a temperature of about 22 °C Part of soil sample (approx.. 100 g) was separated to determine granulometric composition by the aerometric method of Casagrande, modified by Prószyński (Warzynski, Sosnowska & Harasimiuk, 2018). The rest of the soil samples were grounded in a mortar and sieved through one mm mesh sieves to determine soil pH by the potentiometric method in H_2_O and 1N KCl andorganic carbon (OC) by Shimadzu TOC-5000A apparatus at 680 °C.

### PCB extraction of soil, plant and water sample

The air-dried soils were grounded in a mortar and sieved through one mm sieve. The plants’ samples were air-dried and following prior fragmented in the grinder, in the above-ground parts and, in the case of *Solidago canadensis,* in stems and leaves, separately. Both soil and plant samples were stored in paper bags in room temperature until analysis.

About 15–20 g of dry soil or 5–10 g of dry plant material was extracted in n-hexane (95% pure) using a fast ASE 350 extractor for 20 min in elevated pressure and a temperature of 120 °C. The extract was transferred to a flask and concentrated to 1 ml in a vacuum evaporator with a heated bath at 40°. The 5 ml of n-hexane was added to the concentrated extract. The solution was purified using column chromatography. The glass columns were filled with florosil (five cm - bottom) and aluminium oxide (five cm). Gradient washing out was applied, using 25 ml n-hexane and 10 ml mixture of n-hexane: acetone (max. 5% acetone in the mixture). The eluate was concentrated to dry form in a vacuum evaporator with a heated bath at 55 °C. The remaining substance was dissolved in 1 ml n-hexane (GC 99% pure).

The water samples were stored in the refrigerator at 4 °C until analysis. After the water samples reached room temperature the 1,000 ml of water were measured to glass separators at a capacity of 1 L, then 50 ml dichloromethane was added and extracted. The extract (bottom fraction) was then filtered through a funnel filled with anhydrous sodium sulphate into 100 ml flasks. The process was repeated twice. The extract (100 ml) was concentrated to dry form using a vacuum evaporator with a heated bath at 40 °C. The remaining substance was dissolved in 1 ml n-hexane (GC 99% pure).

### Instrumental analysis of soil, plant and water samples

Such prepared analyte was analyzed using gas chromatography with Varian electron capture detector (GC/ECD). The substances were separated using the VF-Xms column (30 m × 0,25 mm × 0,25 m), helium was applied as the carrier gas (purity 5.0; flow 1 ml/min). The temperature sequence in the oven was as follows: 70 °C for 3 min and 70–300 °C at a rate of 5 °C/min (Gabryszewska, Gworek & Garlej, 2018). Qualitative analysis of the studied compounds was based on signals (peak surface) using the calibration curve method. The limit of quantification (LOQ) was evaluated for all analyzed compounds. LOQ value corresponded to the lowest value of the calibration curve and was converted into a sample weight for each congener and confirmed by analysis of fortified samples at the LOQ level for each sample type. Indicative congeners with expanded uncertainties (U) were determined in the studied samples, their values are presented in per cents. The recoveries were calculated for each congener based on the testing of soil certified materials (Sigma-Aldrich RT Corp Certified reference materials for clay soil and loamy sand) and the spiked samples for plants and water adding the known concentration of standard. Final results for soils and plants were calculated taking into account the recovery values for each congener. Method validation parameters are presented in Table 1.

**Table 1:** Validation parameters.

PCB congener	**PCB 28**	**PCB 52**	**PCB 101**	**PCB 118**	**PCB 138**	**PCB 153**	**PCB 180**
Retention time min	30.3	31.6	34.8	37.2	39.5	38.1	42.5
Linearity: Correlation coefficient R^2^	0.994	0.993	0.993	0.995	0.997	0.997	0.997
LOQ soils (corresponding to lowest level of calibration curve) (µg kg^−1^)	0.007	0.012	0.008	0.006	0.012	0.006	0.004
LOQ plants (corresponding to lowest level of calibration curve) (µg kg^−1^)	0.028	0.013	0.011	0.018	0.012	0.011	0.016
LOQ water (corresponding to lowest level of calibration curve) (µg L^−1^)	0.0008	0.0002	0.0002	0.0002	0.0002	0.0001	0.0003
Precision of certified material soils %RSD *n* = 6	7.04	9.35	3.29	4.49	5.48	5.18	7.70
%Recovery of certified material soils *n* = 6	67.2	67.8	75.5	74.8	76.0	68.5	66.1
Precision of spiked samples plant with 0.1 (µg kg^−1^) *n* = 6	7.62	10.53	8.42	6.60	12.16	10.84	13.8
% Recovery of spiked samples plant with 0.1 (µg kg^−1^) *n* = 6	102.1	72.2	74.5	75.2	66.9	71.5	63.9
Precision of spiked samples water with 0.1 (µg L^−1^) *n* = 6	8.10	6.01	5.72	4.87	4.28	11.57	12.50
% Recovery of spiked samples water with 0.1 (µg L^−1^) *n* = 6	98.5	85,7	86.3	81.4	79.8	78.2	70.3
Uncertainty *k* = 2, *p* = 0.05 (%)	30	23	35	33	27	36	30

### Biological indices

Indices were calculated according to the following formulae (Gworek et al., 2016):

- Biological Accumulation Coefficient (BAC) expresses the ratio of PCBs concentration in plants to its concentration in the soil (0–20 cm) as follows:

BAC = PCB_plant_/PCB_soil(0−20cm)_

- Mobility Ratio (MR) expresses the ratio of PCBs concentration in soil (0–20 cm) to its concentration in groundwater:

MR = PCB_soil(0−20cm)_/PCB_groundwater_.

## Results

### PCBs in soils

The texture of the soils examined is loose sands and weak loamy sands. The organic carbon contents in the upper soil levels ranged from 0.8% to 10% and decreased with the depth of soil profile. Most soils had pH values between 2.8–6.55 (pH in 1N KCl) or 3.36–7.04 (pH in H_2_O), and pH slightly increased with the soil depth (Table 2). The contents of congeners determined in the soils were converted into contents of PCB homologues (Table 2).

**Table 2:** Contents of PCB homologues in the soil along with the standard deviation (µg kg^−1^) ± SD dry weight, organic carbon contents (%OC), *n* = 3.

**Plot No.**	**Depth (cm)**	**Tri-CB**	**Tetra-CB**	**Penta-CB**	**Hexa-CB**	**Hepta-CB**	**∑ PCB**	**%OC**	**pH in KCl**	**pH in H**_**2**_**O**
1	0–20	2.015 ± 0.604	1.573 ± 0.314	0.62 ± 0.131	5.904 ± 1.358	0.053 ± 0.010	10.169	10.044	3.67	3.75
	20–50	0.788 ± 0.205	0.591 ± 0.154	0.217 ± 0.046	2.354 ± 0.699	<0.017	3.956	0.677	2.83	3.17
	50–80	0.163 ± 0.041	0.235 ± 0.061	0.054 ± 0.017	0.106 ± 0.022	<0.017	0.575	0.028	4.3	4.76
3	0–20	0.663 ± 0.172	0.418 ± 0.109	0.142 ± 0.043	3.345 ± 0.803	<0.017	4.585	0.823	3.79	4.08
	20–50	0.349 ± 0.091	0.276 ± 0.066	0.076 ± 0.019	0.079 ± 0.18	<0.017	0.797	0.035	4.61	4.95
	50–80	–	–	–	–	–	–			
7	0–20	0.524 ± 0.147	1.300 ± 0.299	0.580 ± 0.151	0.1809 ± 0.049	<0.017	2.610	5.064	5.82	5.91
	20–50	0.959 ± 0.249	0.596 ± 0.155	0.139 ± 0.032	0.024 ± 0.007	<0.017	1.735	0.135	6.55	7.04
	50–80	–	–	–	–	–	–	-	-	-
8	0–20	0.279 ± 0.075	0.413 ± 0.109	0.098 ± 0.025	0.628 ± 0.163	<0.017	1.435	1.577	2.8	3.36
	20–50	0.206 ± 0.053	0.211 ± 0.061	0.076 ± 0.018	0.053 ± 0.014	<0.017	0.563	0.027	3.4	3.56
	50–80	0.380 ± 0.095	0.406 ± 0.105	0.149 ± 0.041	0.078 ± 0.019	<0.017	1.030	0	3.97	4
9	0–20	1.196 ± 0.129	0.781 ± 0.195	0.144 ± 0.040	0.936 ± 0.271	<0.017	3.074	1.505	3.21	3.49
	20–50	0.461 ± 0.134	0.965 ± 251	0.097 ± 0.23	0.063 ± 0.013	<0.017	1.604	0.336	3.74	3.77
	50–80	–	–	–	–	–	–	0.104	4.22	4.26
10	0–20	0.723 ± 0.188	0.703 ± 0.2018	0.414 ± 0.103	0.152 ± 0.036	<0.017	2.009	9.622	5.45	5.75
	20–50	–	–	–	–	–	–	–	–	–
	50–80	0.245 ± 0.071	0.309 0.074	0.111 ± 0.029	0.027 ± 0.007	<0.017	0.709	0.494	5.99	6.14

**Notes.**

< detection limit.

– not detected.

### PCBs in plants

**Figure 2:**
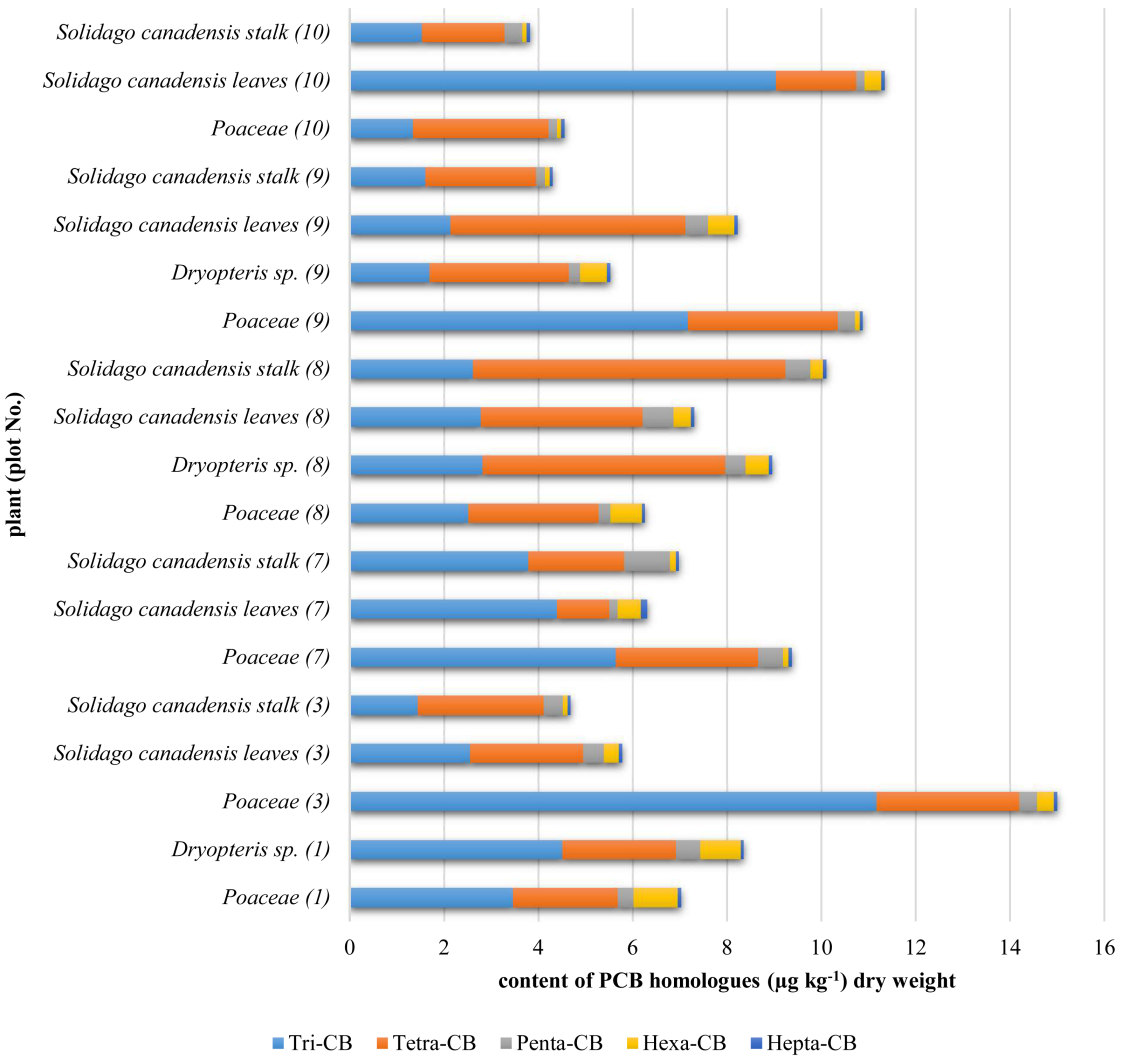
PCB homologues contents in plants.

Contents of congeners determined in plants were converted into contents of PCB homologues and presented in Fig. 2.

### PCBs in water

**Figure 3:**
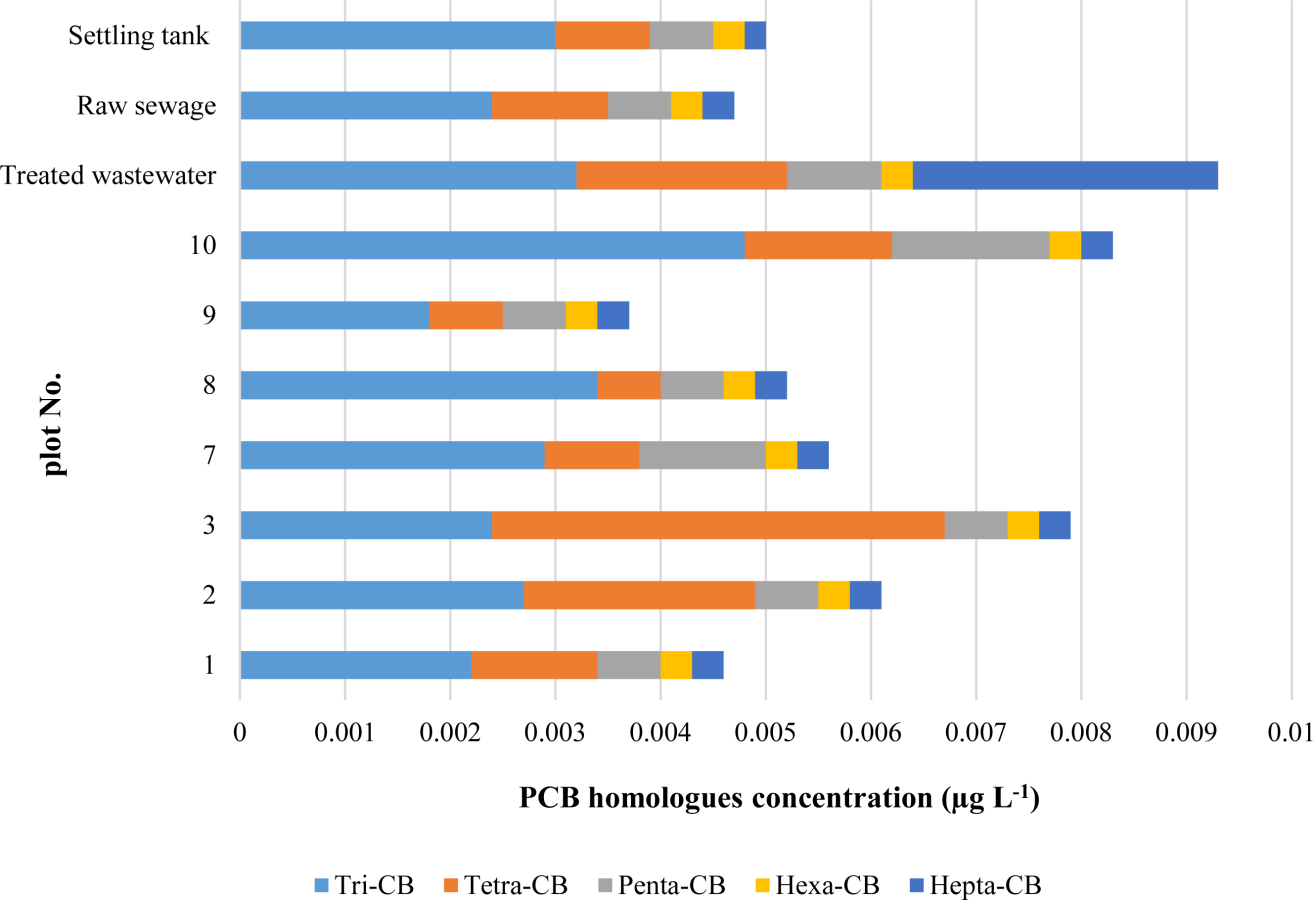
Concentrations of PCB homologues in waters.

The PCBs content in groundwater is shown in Fig. 3. The study area 9 was selected for the assessment of water pollution by PCBs as an area outside the impact of the landfill.

**Figure 4:**
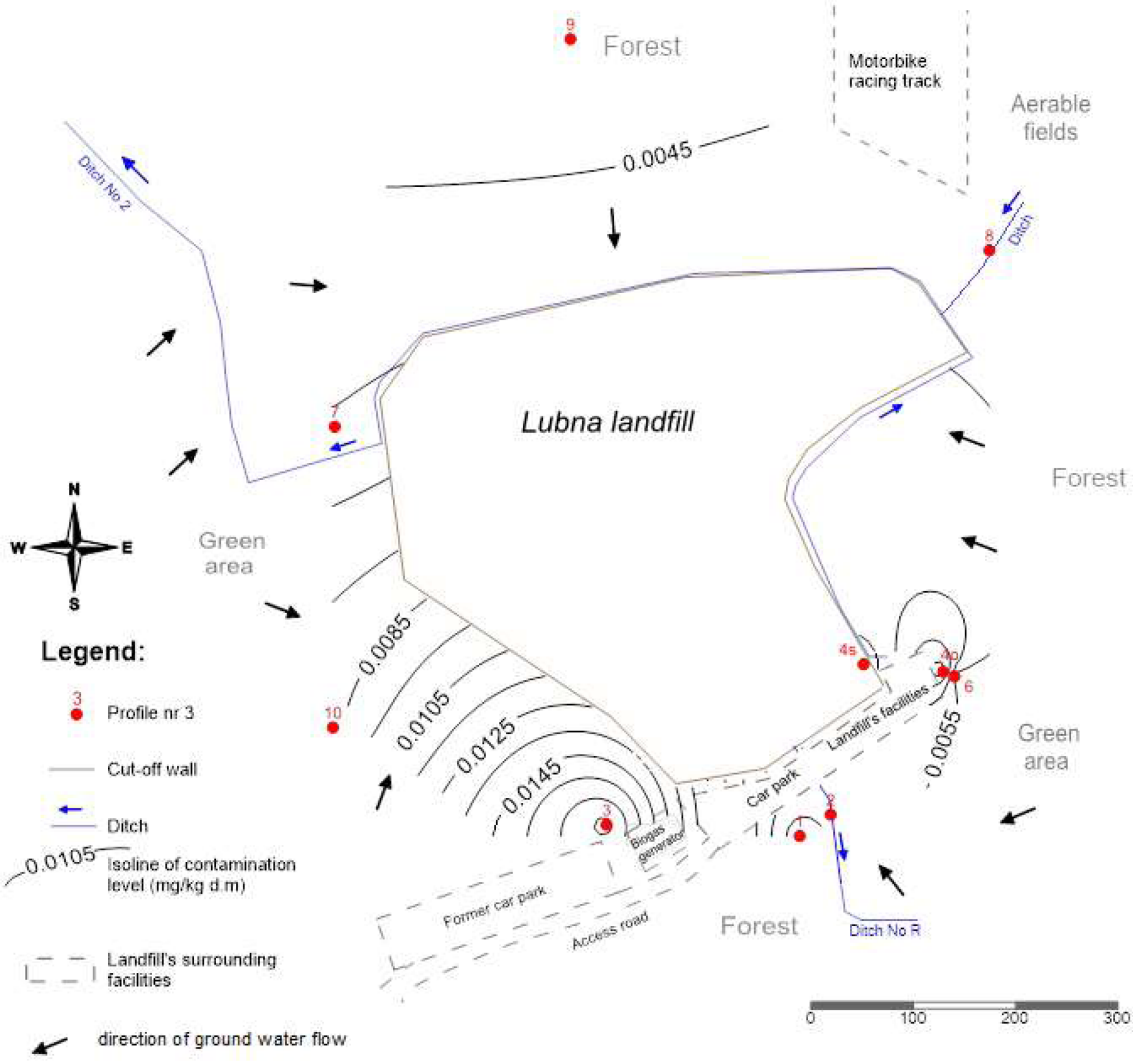
Spatial distribution of PCBs contents in groundwater.

This plot is located in the direction of the water flow towards the landfill site, and the concentrations of PCBs homologues were there smallest as compared to the concentrations found in the remaining plots (Fig. 4). Figure 4 shows the spatial distribution of PCBs concentrations in groundwater using isolines.

### Indicators

**Figure 5:**
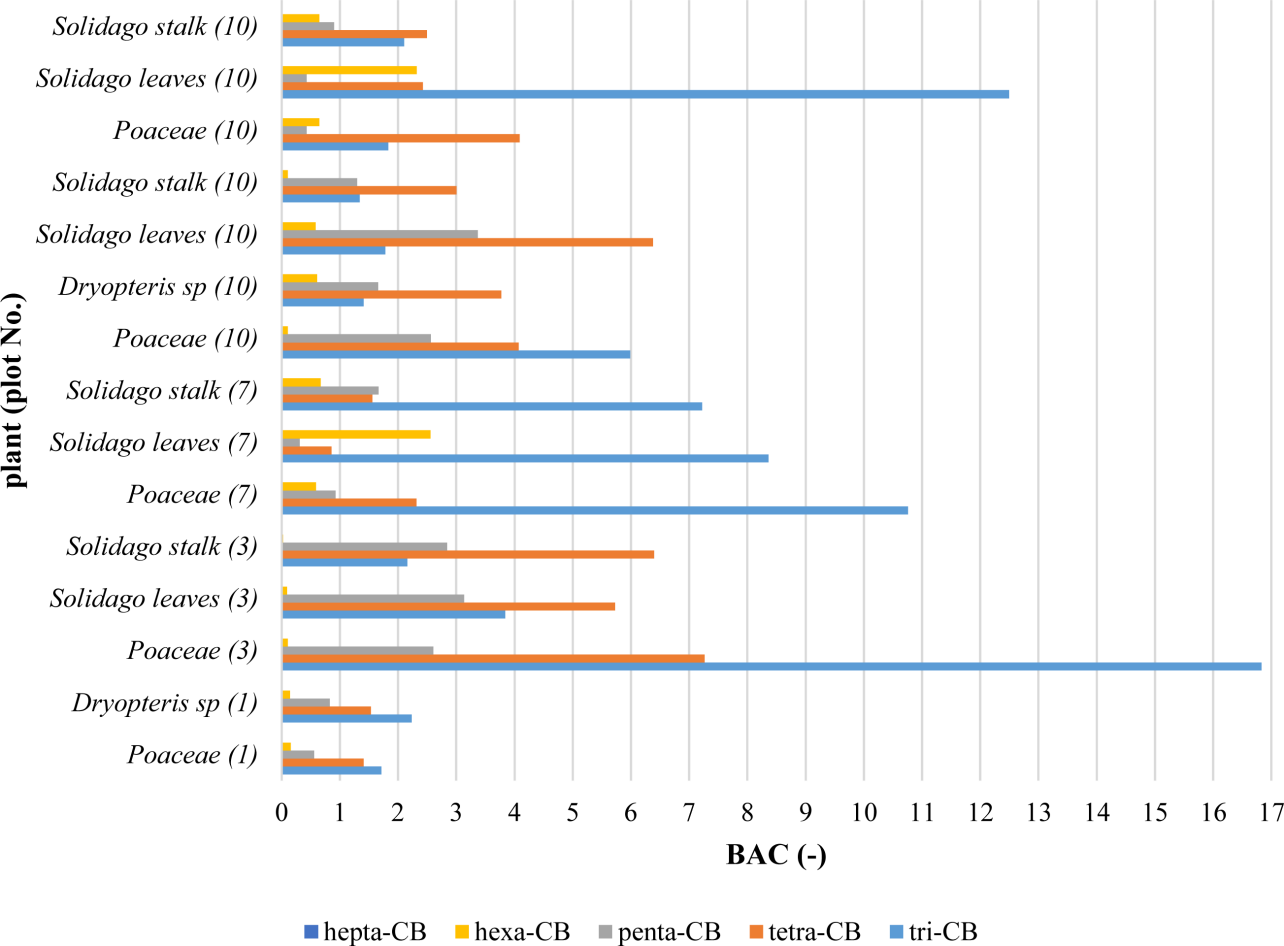
Values of PCBs bioaccumulation coefficients (BAC) in plants.

The hepta-CB contents in plants and the soil, in most cases, were below the detection limit. For the calculation of BAC and MR coefficients, no account was taken of the results below the determination limit. Values of BAC coefficients were presented in Fig. 5. The calculated values of PCBs migration coefficients from the soil to groundwater (Table 3) shown the soil contamination impact on PCBs water concentration.

## Discussion

### PCBs in soils

According to the soil’s granulometric composition, soils were built from loose sands and loamy sand. According to Polish law ( Minister of the Environmet of Poland, 2016), no values, of the tested PCB congeners, exceeded the threshold value of 20 μg kg^−1^. Homologues tri-CB and tetra-CB were found to attain the highest contents in the soils (Table 2). These elevated contents may be due to the fact that PCBs molecules with a larger number of chlorine atoms are possibly broken down to lower homologues by reductive dechlorination (WHO, 1992). It was noted that the penta-CB and hexa-CB homologues were transferred to a depth of 30 cm, whereas the tri-CB and tetra-CB homologues showed the capacity to migrate to the deeper soil layers. This is a consequence of the fact that the lower homologues have lower octanol-water partition coefficients (Larry & Larry, 2001) than the higher homologues and, consequently, the sorption in the soil is weaker and the possibility of migrating down the soil profile is greater. This may be due to the translocation of the smallest soil particles along with the absorbed PCBs, which can be explained by the soil texture (loose sands and weak loamy sands) and or by leaching with rainwater deeper into the soil.

Figure 1 shows the spatial distribution of the sum of the PCBs contents determined around the landfill for the soil layer: 0–20 cm (with the highest PCBs contents). Additionally, water flow directions are marked in Fig. 1. The effect of water flow direction on the soil PCBs contents is not perceptible due to the fact that the landfill is situated on the water division. Also, the poor water solubility of PCBs contributes to a weak migration of PCBs with water in the soil. The highest PCBs contents were found in the topsoil (0–20 cm). The highest concentrations in the soil were found for the 138 (hexa-CB) congener.

The highest PCBs contents were determined in the plots Nos. 1 and 3, which were located close to the entrance to the landfill site. In addition, there was a car park and a biogas generator nearby. The higher soil contents of PCBs found for these plots result from an additional source of pollutants such as exhausts from road transport (Gabryszewska, Gworek & Garlej, 2018). The sum of PCBs contents in the topsoil was within the range of 2.0–24.5 µg kg^−1^. In the topsoil layer (0–15 cm) around municipal landfills in Havana, the PCBs contents were below 0.05 mg kg^−1^ (Espinosa Lloréns et al., 2008). In a study by Melnyk and others on the PCBs contents in the topsoil (0–20 cm) around the Gdańsk landfill in Poland, the average PCBs contents in the soil were 4.5 μg kg^−1^ (Melnyk et al., 2015).

This study provided evidence that the contents of a hexa-CB homolog in the soil decreased along with the distance from the landfill. The highest contents of PCBs in the soil were determined at a distance of 50 m from the site.

### PCBs in plants

The highest PCBs contents were determined in plants from the *Poaceae* family as compared to those found in *Solidago canadensis* and *Dryopteris* sp. (Fig. 2). It *Poaceae* have a very extensive root system, they form turf, which enables the grasses to sustain large amount contamination (Kozlowski, 2012). Grassroots hairs are found in the upper soil layer where there are the largest contents of PCBs. *Solidago* has a pile root system, and roots hair are located at the end of the main root, i.e., deep in the soil where the PCBs contents were negligible. *Dyropters* has adventitious roots and a significant part of them is deposited on the surface but they are shielded mostly by fern leaves what limits PCBs deposition on soil and then the uptake of PCBs.

The sums of the PCB homologues contents in plants were as follows: *Poaceae*—4.5–15.0 μg kg^−1^; *Solidago canadensis* leaves—5.7–11.3 μg kg^−1^, *Solidago canadensis* stalks—3.8–10.1 μg kg^−1^ and *Dryopteris* sp.—5.5–8.9 μg kg^−1^. The *Poaceae* plants from the plot No 3 were found to contain the highest amounts of PCBs, likewise as soils from this plot, which was affected by the road transport as an additional source of pollution (Gabryszewska, Gworek & Garlej, 2018). The PCBs contents in leaves of *Solidago canadensis* were, in most cases, higher than contents in their stalks, indicating that air deposition was most likely the main source of pollution. Leaves have a larger area than stems and are covered with wax which with high Kow coefficients for PCBs could cause that PCBs contents in the leaves were higher than in the plant stems. In the leaves much higher PCB 138 (hexa-CB) content was detected than in the stems. Such large compounds are not transferred from the roots to the stem and leaves, which clearly indicates an additional source of PCBs in the leaves, which is the PCBs deposition from the air. The evidence from a field study with *Cucurbita pepo* grown on soil with average PCBs content of 21 μg g^−1^, as the unique contamination source, indicated that the PCBs contents in plant stalks were higher (11 μg g^−1^) than that in leaves (8.9 μg g^−1^) (Whitfield Åslund et al., 2008). Chem et all indicated that the large agglomeration like Dalian in China influences PCBs contents in plants, the average PCBs contents in needles (*Cedrus deodara*) were 4.4 ± 1.5 μg g^−1^ (Chen et al., 2006). The study conducted in China did not show any dependence between the PCBs contents in plants and the distance from the shutter or the direction of water flow (Chen et al., 2006).

PCBs with 7 chlorine atoms in the molecule has a high partition coefficient and are less accessible for plants via soil, hence the hepta-CB contents in the plants were the lowest as compared to the other homologues. The most common source of hepta-CB and octa-CB in plants is dry deposition (Böhme, Welsch-Pausch & McLachlan, 1999).

**Table 3:** The values of PCB migration coefficients from soil to groundwater. Not calculated due to not detected PCBs in soil.

Plot/point No.	MR (-)				
	tri-CB	tetra-CB	penta-CB	hexa-CB	hepta-CB
1	915.9	1310.8	–	–	–
3	276.3	97.2	–	–	–
7	96.2	458.9	81.7	–	–
8	154.1	2166.7	–	–	–
9	664.4	1115.7	–	–	–
10	150.6	502.1	276.0	–	–

The dominant homologues in plants were: tri-CB and tetra-CB. In most cases, hexa-CB homologue was found to occur with the highest contents in the topsoil layer. Whereas, the content of this homologue in plants was insignificant, what may be understood that it was not taken up from the soil. The highest levels of tri-CB and tetra-CB were determined in both plants and the topsoil. This may testify to the fact that, in addition to the uptake of PCBs from the soil, the other probable source of these compounds for plants was probably the deposition from the air. In the hydroponic cultivation of poplars, Liu has experimentally shown that the PCBs compounds were taken up by plants and translocated. In the roots, there were detected the compounds with 1 chlorine atom (congener 3), with 2 chlorine atoms (congener 15) and with 3 (congener 28) and 4 chlorine atoms (congeners 52 and 77). Translocation to the stem was observed for mono-, di- and tri-CB compounds. However, this phenomenon was not acknowledged for the compounds with four chlorine atoms. No PCBs were detected in the leaves (Liu & Schnoor, 2008).

The highest concentration of tri-CB was determined in water sampled from the plots Nos. 7, 8 and 10 (Fig. 3). The elevated concentrations of low chlorinated PCBs found at these plots may be explained by a relative closeness of the plots to the landfill and by the possibility of PCB migration along with the direction of water flow (from N to SW). With the assumption that 1 L of water is 1 kg, it can be said that the concentrations of PCBs homologues in water were on average 400 times lower than their contents in soils. As was previously mentioned, on the occasion of discussing the soil results, PCBs are strongly absorbed by organic matter due to the high octanol-water partition coefficients, and under favourable conditions, can be leached into waters (Erickson, 2001). Also, PCB are slightly soluble, PCB higher chlorinated have lower solubility than PCB with a small number of chlorine atoms. Therefore concentrations of tri-CB and tetra-CB in water were higher than the concentration of the rest homologues.

Considering the direction of water flow (Fig. 4), water sampled from the study plot No. 9 was assumed as the reference for groundwater. It was noted that only compounds with three and four chlorine atoms were migrating. The total concentration of PCBs concerning the reference PCBs concentration (soil from research area 9) increased by 2.5 - plot No. 10; 1.4 –plot No. 7; 191,7 –plot No. 3; 1,2 –plot No. 1. The highest concentrations in groundwater were determined on plot No. 3, 58 m from the landfill. This concentration was 192 times higher than the concentration in water from the reference object (No. 9). It was also noted that PCBs concentrations in water were not dependent on the distance from the landfill. As in the case of soils and plants, higher concentrations were influenced by an additional source of pollution (transport).

The PCBs compounds with 3 chlorine atoms in the molecule were dominating in groundwater and among them congeners 18 and 28. Next to tri-CB, in the surface waters, there was a significant share of tetra-CB. The highest total concentration found in groundwater was 0.0179 μg L^−1^. The PCBs in the amount of 0.01–3.1 μg L^−1^ were reported in leachates from municipal waste landfills in Norway (Haarstad & Borch, 2008), which exceeded the concentrations determined around the landfill examined.

There was a higher concentration of PCBs with 3, 4, 5 and 7 chlorine atoms in the treated than in the raw sewage, which results from the fact that sewage purification does not remove PCBs (Fig. 3). In the biological wastewater treatment, microorganisms decompose mono-, di- and tri-chlorobiphenyls relatively quickly, while tetra-chlorobiphenyls - relatively slowly. Biphenyls with a higher degree of chlorination are assumed to be resistant to biodegradation (WHO, 1992).

### Indicators

It is assumed that compounds for which the bioaccumulation factor is greater than 1 are accumulated (Whitfield Åslund et al., 2008). Values of BAC coefficients (Fig. 5) have shown limited possibilities of taking compounds belonging to penta-CB and hexa-CB homologues. In the case of tri-CB and tetra-CB homologues, the values of BAC coefficients significantly exceeded the value of 1, but the contents of the above compounds in the soil and plants indicated that the contamination in the landfill site was low and at the reference level. Studies carried on aquatic plants indicated that *Nelumbo nucifera* spp. can accumulate PCBs by root uptake, probably by biotransformation within plant tissues (Dai et al., 2014). In China, it was discovered that the elevated PCBs levels in mangrove leaves may be caused by atmospheric sedimentation. The biota sediment accumulation factor for PCBs was 9.9, indicating mangroves’ ability to bioaccumulation of PCBs (Qiu et al., 2019).

The high values of coefficients of the PCBs migration from the soil to groundwater (Table 3) point out to an insignificant impact of the PCBs contents in soils on water pollution. The values of the tri-CB migration coefficients are many times lower than the values of tetra-CB coefficients due to physicochemical properties of these homologues. The water solubility of compounds derived from the tri-CB homologue is greater than the solubility of the PCBs belonging to the tetra-CB homologue. Also, the values of octanol-water partition coefficients for tetra-CB are higher than for tri-CB, hence the compounds belonging to the tetra-CB homologue are subject to stronger sorption in the soil and are less eluted than the tri-CB compounds.

## Conclusions

The two PCBs homologues, i.e., tri-CB and tetra-CB dominated in the soils within the zone of influence of the municipal waste landfill. The reason for this is the biological decomposition of PCBs compounds with a large number of chlorine atoms by separating the chlorine atoms from them. In addition, a significant part of the composition of industrial PCBs mixtures were low-chlorinated PCBs congeners. The largest accumulation of PCBs compounds was determined in the topsoil layers. The ability of PCBs compounds to move deep into the soil to a depth of about 30 cm was observed. The highest accumulation of hexa-CB (mainly PCB 138) was determined in the topsoil layers. Due to the high value of the octanol-water partition coefficient, PCB 138 was strongly sorbed in the topsoil layer and did not move deep into the soil. The observed influence of the landfill distance on the accumulation of hexa-CB in the soil. The accumulation of hexa-CB in the soil decreased with increasing distance from the landfill.

In plants, similarly to soil, tri-CB and tetra-CB homologues dominated. Due to the lower octanol-water partition coefficient, these compounds are more mobile and can be more easily absorbed by plants from the soil. The accumulation of tri-CB and tetra-CB compounds in plants was confirmed by calculating the bioaccumulation coefficient (BAC). The BAC values for tri-CB and tetra-CB were higher than 1. The largest accumulation of PCBs occurred in Poaceae. This is due to the extensive root system and large leaf area concerning the whole plant.

The total PCB content in the first aquifer was in the range 0.0037–0.0179 μg L-1. Assuming that 1 L of water is 1 kg, the PCBs content in water was significantly lower than in soils and plants. The influence of groundwater flow direction on PCBs concentration in groundwater was observed. The concentration of low-chlorinated PCBs in water increased with the direction of water outflow from the landfill (from N to SW). No influence of the groundwater flow direction on the PCBs accumulation in soils and plants was found. High values of PCBs migration coefficients (MR) from soil to groundwater showed the insignificant influence of PCBs content in soil on water pollution.

##  Supplemental Information

10.7717/peerj.10546/supp-1 Supplemental Information 1 PCBs content.

## Additional Information and Declarations

### Field Study Permissions

The following information was supplied relating to field study approvals (i.e., approving body and any reference numbers): Institution: “Municipal waste landfill Łubna”, Eugeniusz Koda, has given oral consent for sampling. Nowadays this landfill is closed and after remediation.

### Data Availability

The following information was supplied regarding data availability: Raw data, including all PCBs content in the soil, plants, and water, are available as a Supplemental File.
