# Supplementary material for: Municipal waste landfill as a source of polychlorinated biphenyls releases to the environment
Source: PeerJ. 2021 Jan 15;9:e10546. doi: 10.7717/peerj.10546 (PMC7812931; doi:10.7717/peerj.10546)
Supplement: Supplemental Information 1 [file peerj-09-10546-s001.docx]

**Table S1.** PCBs content in soil (μg kg^-1^)

| **Plot No.** | **Depth (cm)** | **PCBs congeners** | | | | | | |
| --- | --- | --- | --- | --- | --- | --- | --- | --- |
|  |  | **28** | **52** | **101** | **118** | **153** | **138** | **180** |
| 1 | 0-8 | 0.406 | 2.126 | 0.318 | 0.195 | < 0.006 | 16.019 | < 0.017 |
|  | 8-15 | < 0.005 | 1.613 | 0.077 | 0.125 | 0.116 | 2.264 | 0.147 |
|  | 15-25 | < 0.005 | 0.114 | < 0.010 | < 0.010 | < 0.006 | 0.184 | < 0.017 |
|  | 25-35 | 0.017 | 0.071 | 0.034 | < 0.010 | < 0.006 | < 0.008 | < 0.017 |
|  | >80 | 0.014 | 0.064 | < 0.010 | < 0.010 | 0.088 | < 0.008 | < 0.017 |
| 3 | 0-20 | 0.032 | 0.245 | < 0.010 | < 0.010 | < 0.006 | 3.318 | < 0.017 |
|  | 40-50 | < 0.005 | 0.118 | < 0.010 | < 0.010 | 0.061 | < 0.008 | < 0.017 |
| 7 | 0-25 | < 0.005 | 0.920 | < 0.010 | < 0.010 | < 0.006 | 0.168 | < 0.017 |
|  | 25-50 | < 0.005 | 0.254 | 0.066 | < 0.010 | < 0.006 | < 0.008 | < 0.017 |
| 8 | olf | 0.345 | 1.584 | 0.198 | < 0.010 | < 0.006 | 1.482 | < 0.017 |
|  | 0-20 | 0.053 | 0.232 | 0.030 | < 0.010 | 0.097 | 0.521 | < 0.017 |
|  | 20-40 | 0.027 | 0.106 | 0.032 | < 0.010 | < 0.006 | 0.034 | < 0.017 |
|  | 55-80 | < 0.005 | 0.164 | < 0.010 | < 0.010 | < 0.006 | 0.062 | < 0.017 |
| 9 | olf | < 0.005 | 0.536 | < 0.010 | < 0.010 | < 0.006 | 1.393 | < 0.017 |
|  | 0-20 | 0.148 | 0.404 | < 0.010 | < 0.010 | 0.104 | 0.822 | < 0.017 |
|  | 20-25 | 0.054 | 0.096 | < 0.010 | < 0.010 | < 0.006 | 0.092 | < 0.017 |
|  | 25-32 | 0.073 | 1.575 | < 0.010 | < 0.010 | < 0.006 | 0.041 | < 0.017 |
|  | 32-42 | 0.042 | 0.638 | < 0.010 | < 0.010 | < 0.006 | < 0.008 | < 0.017 |
| 10 | 0-20 | 0.059 | 0.427 | 0.277 | < 0.010 | < 0.006 | 0.111 | < 0.017 |
|  | >40 | 0.045 | 0.234 | < 0.010 | < 0.010 | < 0.006 | < 0.008 | < 0.017 |

**Table S2.** PCBs content in plant (μg kg^-1^)

| **Plot No.** | **Plant** | **PCBs congeners** | | | | | | |
| --- | --- | --- | --- | --- | --- | --- | --- | --- |
|  |  | **28** | **52** | **101** | **118** | **153** | **138** | **180** |
| 1 | *Poaceae* | 0.112 | 2.124 | < 0.038 | < 0.040 | < 0.025 | 0.872 | < 0.070 |
|  | *Dryopteris* sp. | 0.822 | 2.316 | < 0.038 | < 0.040 | < 0.025 | 0.787 | < 0.070 |
| 3 | *Poaceae* | 0.338 | 2.388 | 0.231 | < 0.040 | 0.046 | 0.277 | < 0.070 |
|  | *Solidago canadensis* leaves | 0.468 | 2.007 | 0.117 | < 0.040 | < 0.025 | 0.202 | < 0.070 |
|  | *Solidago canadensis* stalk | 0.406 | 1.909 | 0.265 | < 0.040 | < 0.025 | < 0.033 | < 0.070 |
| 7 | *Poaceae* | 0.198 | 2.010 | < 0.038 | < 0.040 | < 0.025 | < 0.033 | < 0.070 |
|  | *Solidago canadensis* leaves | 0.965 | 0.602 | < 0.038 | < 0.040 | < 0.025 | 0.415 | 0.138 |
|  | *Solidago canadensis* stalk | 0.183 | 1.205 | 0.082 | < 0.040 | < 0.025 | < 0.033 | < 0.070 |
| 8 | *Poaceae* | 0.212 | 1.730 | < 0.038 | < 0.040 | < 0.025 | 0.601 | < 0.070 |
|  | *Dryopteris* sp. | 0.333 | 3.841 | < 0.038 | < 0.040 | < 0.025 | 0.436 | < 0.070 |
|  | *Solidago canadensis* leaves | 1.029 | 1.821 | < 0.038 | 0.389 | < 0.025 | 0.306 | < 0.070 |
|  | *Solidago canadensis* stalk | 0.435 | 4.937 | < 0.038 | < 0.040 | < 0.025 | 0.204 | < 0.070 |
| 9 | *Poaceae* | 0.537 | 2.524 | < 0.038 | < 0.040 | < 0.025 | < 0.033 | < 0.070 |
|  | *Dryopteris* sp. | 0.425 | 1.462 | < 0.038 | < 0.040 | < 0.025 | 0.506 | < 0.070 |
|  | *Solidago canadensis* leaves | 1.140 | 4.002 | < 0.038 | < 0.040 | < 0.025 | 0.484 | < 0.070 |
|  | *Solidago canadensis* stalk | 0.249 | 1.827 | < 0.038 | < 0.040 | < 0.025 | < 0.033 | < 0.070 |
| 10 | *Poaceae* | 0.431 | 1.198 | < 0.038 | < 0.040 | < 0.025 | < 0.033 | < 0.070 |
|  | *Solidago canadensis* leaves | 0.647 | 0.798 | < 0.038 | < 0.040 | < 0.025 | 0.288 | < 0.070 |
|  | *Solidago canadensis* stalk | 0.240 | 1.135 | < 0.038 | 0.236 | < 0.025 | < 0.033 | < 0.070 |

**Table S3.** PCBs concentration in water (μg L^-1^)

| **Plot No.** | **PCBs congeners** | | | | | | |
| --- | --- | --- | --- | --- | --- | --- | --- |
|  | **28** | **52** | **101** | **118** | **153** | **138** | **180** |
| 1 | 0.0008 | 0.0003 | < 0.0002 | < 0.0002 | < 0.0001 | < 0.0002 | < 0.0003 |
| 2 | 0.0010 | 0.0018 | < 0.0002 | < 0.0002 | < 0.0001 | < 0.0002 | < 0.0003 |
| 3 | 0.0010 | 0.0031 | < 0.0002 | < 0.0002 | < 0.0001 | < 0.0002 | < 0.0003 |
| Treated wastewater | 0.0008 | 0.0006 | < 0.0002 | < 0.0002 | < 0.0001 | < 0.0002 | 0.0029 |
| Raw wastewater | 0.0011 | 0.0003 | < 0.0002 | < 0.0002 | < 0.0001 | < 0.0002 | 0.0003 |
| Secondary settling tank | 0.0009 | < 0.0002 | < 0.0002 | < 0.0002 | < 0.0001 | < 0.0002 | 0.0002 |
| 7 | 0.0008 | 0.0002 | < 0.0002 | < 0.0002 | < 0.0001 | < 0.0002 | 0.0003 |
| 8 | 0.0027 | 0.0002 | < 0.0002 | < 0.0002 | < 0.0001 | < 0.0002 | < 0.0003 |
| 9 | 0.0009 | < 0.0002 | < 0.0002 | < 0.0002 | < 0.0001 | < 0.0002 | < 0.0003 |
| 10 | 0.0029 | 0.0003 | < 0.0002 | < 0.0002 | < 0.0001 | < 0.0002 | 0.0003 |
